# Analysis of Cuticular Lipids of the Pharaoh Ant (*Monomorium pharaonis*) and Their Selective Adsorption on Insecticidal Zeolite Powders

**DOI:** 10.3390/ijms19092797

**Published:** 2018-09-17

**Authors:** Heleen Van Den Noortgate, Bert Lagrain, Tom Wenseleers, Johan A. Martens

**Affiliations:** 1 Centre for Surface Chemistry and Catalysis, KU Leuven, Celestijnenlaan 200F, Box 2461, 3001 Heverlee, Belgium; heleen.vandennoortgate@biw.kuleuven.be (H.V.D.N.); bert.lagrain@kuleuven.be (B.L.); 2 Lab of Socio-Ecology & Social Evolution, KU Leuven, 3000 Leuven, Belgium; tom.wenseleers@kuleuven.be

**Keywords:** pest management, zeolites, wax adsorbents, pharaoh ants

## Abstract

The pharaoh ant is a notorious and hard to eradicate pest, which poses a threat in hospitals, spreading pathogens and contaminating sterile equipment. When applied on ants, zeolites adsorb part of their epicuticular wax layer. The ants are then vulnerable to desiccation, since this layer regulates water exchange. We analyzed the chemical composition of this wax layer using GC-MS (Gas Chromatography-Mass Spectrometry). A hexane wash of *M. pharaonis* foragers resulted in the identification of 53 components, four of which were not previously defined in *Monomorium* species. Selective adsorption of specific compounds on zeolites assisted in the identification of compounds which could not be separated on the GC column and allowed for the identification of three additional compounds. Zeolites show different affinities for the wax compounds depending on pore structure and chemical composition. Selective adsorption of alkanes on zeolites is also investigated in the fields of refinery processes and catalysis. Pore mouth and key lock adsorption mechanisms and selectivity according to molecular weight and branching, investigated in these fields, are also involved in adsorption processes of epicuticular waxes. The insecticidal activity of a zeolite is related to adsorption selectivity rather than capacity. One of the best adsorbing zeolites showed limited insecticidal activity and can be considered as a non-lethal alternative for epicuticular wax sampling.

## 1. Introduction

In previous research, we investigated the insecticidal effectiveness of several powdered materials against the pharaoh ant, a notorious, almost cosmopolitan pest. There is a little controversy about the original habitat of the pharaoh ant, being either Africa [1] or the East Indies [2], however, in both regions, it resides outside in a tropical climate. When the pharaoh ant arrived in countries with a milder climate, it adapted to its new environment by settling in heated buildings, usually in hard-to-reach places such as wall crevices and voids [2,3]. Because of its small size (2 mm) it can even be found inside clean rooms and electrical equipment where it can easily contaminate or destroy instruments [2]. It also poses a severe threat in hospitals by transferring pathogens and contaminating sterile equipment and rooms [3].

Pharaoh ants are social insects with a unicolonial lifestyle. Pharaoh ant colonies can contain vast amounts of individuals and hundreds of queens. Due to this construct, they are extremely hard to eradicate [4,5,6,7] and, on top of that, disturbing a pharaoh ant colony leads to budding. Budding is the phenomenon where a part of the colony moves away from the nest and sets up a new (sub)colony in an alternative location [5,6]. This newly formed (sub)colony often remains in contact with the mother colony, sharing food and other resources, but they can also survive separately [8]. For this reason, when dealing with pharaoh ant infestations, it is best to target the entire population at once.

The approach tested in previous research comprised the use of powdered materials, which upon contact with the ants, extract wax molecules from the epicuticular wax layer, leaving them vulnerable to desiccation [9,10,11,12] because the epicuticular wax layer, together with the rest of the cuticle, is responsible for many bodily functions among which water exchange [13,14,15]. The cuticle of many insects consists of different layers, each with their own composition, and its makeup changes over time as the insect matures or when there are significant changes in the environment. The ability of insects to adapt even to extreme conditions (e.g., heat and drought) stems largely from the ability of the cuticle to change its characteristics, depending on environmental conditions [13]. The epicuticular wax layers of many insects, including several ant species have already been investigated. They reveal very different compositions for different insect types. Some insects’ wax layers contain high amounts of esters or alcohols, others are composed mainly of hydrocarbons [15,16,17,18,19,20].

Our previous investigation revealed that zeolites are among the best performing insecticidal powders [21]. Zeolites are widely used as molecular sieves and catalysts [22]. Due to their specific shape selectivity, they can be used to selectively adsorb certain molecules from a mixture and in catalysis they favor formation of specific isomers, which fit best within the void volumes inside the zeolite crystals [22,23]. In the literature on adsorption and catalysis, molecular shape selectivity is investigated with molecules with up to 24 carbon atoms [23,24]. Since epicuticular hydrocarbons roughly range from eicosane (C_20_) to tetracontane (C_40_), an adsorption study of epicuticular hydrocarbons could reveal whether the adsorption selectivity observed with shorter model molecules is confirmed with longer ones.

This manuscript focuses on the composition of the epicuticular wax layer of the pharaoh ant and the adsorption of its compounds on adsorbent zeolite powders. We show that zeolites depending on their pore structure and pore size are able to selectively adsorb biomolecules according to molecular weight and skeletal branching. This selective adsorption process is useful in the chromatographic analysis because minority compounds with overlapping GC peaks can be made visible by selective adsorption of the masking overlapping compounds in the wax mixture. Since a specific compound in this epicuticular wax layer might have an additional function to water exchange, a complete list of components present in the epicuticular wax layer can be of great interest. (*Z*)-9-pentacosene, for example, is a component present in the wax layer of the locust borer, *Megacyllene robiniae*, and acts as a contact sex pheromone [25]. The presence of minor components in the epicuticular wax layer can also provide information about the synthesis pathways of the epicuticular wax components [26] and, in plant epicuticular wax layers, the minor components can be crucial in determining the crystalloids present on the plant surface [27]. However, the results pertaining to the selectivity of the zeolites are not only useful in the field of biology, but also in the fields of refinery processes and catalysis. In the production of synthetic lubricants, for example, hydrocarbons in the range of C_20_ to C_40_ are the main components and performance is greatly influenced by the exact composition of the lubricant, the molecular weight and branching of the different components [23,28,29]. The same holds for the production of middle distillate fuels, where the cetane number and cold flow properties of the fuels depend strongly on the amount of branching of the molecules and the length of the branches [30].

## 2. Results and Discussion

### 2.1. Hexane Wash of the Epicuticular Wax of M. pharaonis

**Figure 1:**
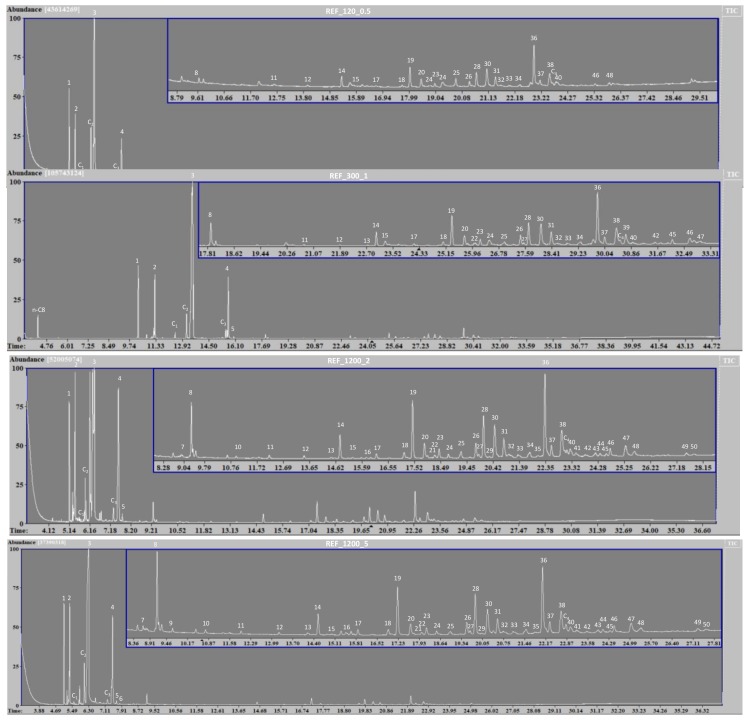
GC-MS spectra of reference samples produced with different amounts of foragers and varying vortex times. All identified peaks are numbered and the corresponding identified compounds are listed in Table 1. Compounds found over different samples carry the same number in every sample. Unidentified peaks were either contaminants (e.g., phtalate compounds from the plastic containers used to contain the ants) or unidentifiable compounds.

Analysis of the hexane wash of the foragers revealed 34 identifiable peaks for the sample using 120 ants and 0.5 min of vortexing (Ant_ref_1). When increasing the number of foragers to 300 (Ant_ref_2), 1200 (Ant_ref_3) and 1200 (Ant_ref_4), the number of peaks identified increased to 40, 51 and 53, respectively. From each peak (Figure 1), one or multiple compounds were identified, which are all listed in Table 1.

The 19 extra peaks identified in Ant_ref_4 sample compared to Ant_ref_1 corresponded mostly to compounds with less than 23 or over 31 carbon atoms and all of these compounds were present in low quantities. Nearly all of the GC peaks, identified in reference samples prepared with more foragers and longer vortex times, already showed corresponding peaks in the other reference samples but the resolution was not sufficient for undoubted identification.

While the n-hexane extracts of the *M. pharaonis* foragers were made to identify the compounds of the epicuticular wax layer, they also contained several alkaloid compounds (Figure 1 and Table 1). These components, including monomorines I, II, III and IV, are not present in the epicuticle, but produced by the ants’ abdominal glands [31]. Due to the complete immersion of the foragers in n-hexane, the extraction was not limited to the epicuticle and compounds from these glands were also present in the extracts. All of the known monomorines were identified and the presence of several pyrrolines was also registered; however, there was no trace of the previously determined trail pheromone 5-methyl-3-butyl-octahydroindolizine (faranal) [32]. A compound we did identify in all of the extracts was *trans*-2-(1-hex-5-enyl)-5-(non-8-enyl)-pyrrolidine, an alkaloid which was previously defined in the venom of other *Monomorium* species [31,33]. A trail component previously found in *Solenopsis invicta*, α-farnesene [34], was also found in all of the samples, but present in very small quantities. Finally, both 2-pentyl-5-(1-hex-5-enyl)-1-pyrroline and 2-(1-hexenyl)-5-(hept-6-enyl)-1-pyrroline were also identified based on their GC-MS spectra [31,35,36,37]. While these exact components were not previously identified, a recent publication by Chen et al. identifies several 2,5-dialkenylpyrrolines as well as a 2-alkyl-5-alkenylpyrroline in the venom of *Monomorium minimum* [38]. Using the same interpretation of the mass spectra (Appendix A) enabled the identification of these compounds.

The epicuticular waxes extracted by n-hexane consisted solely of hydrocarbons, with the exception of cholesterol (Figure 1 and Table 1). Cholesterol was one of the sterols already previously detected in the epicuticular wax layer of *Solenopsis invicta* and *richteri* [18].

**Figure 2:**
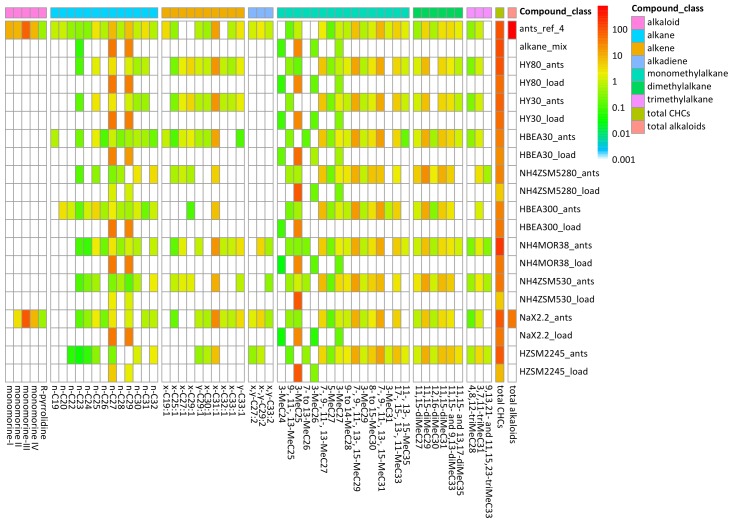
Heat map of the GC-MS data, showing the extracted cuticular profile composition (percent CHC (cuticular hydrocarbons, without alkaloids) and percent alkaloid) of *M. pharaonis* foragers and the adsorption of the different epicuticular compounds by nine zeolite materials upon exposure to foragers for 1 h (represented as _ant). The composition of the alkane mixture (alkane_mix) and the compounds retrieved from the zeolites after loading them with the solution (represented as _load) are also provided. Materials are sorted by induced mortality, from high at the top to low at the bottom [21]. When two compounds were present in the same peak, the most abundant compound was chosen for representation. R-pyrrolidine is *trans*-2-(1-hex-5-enyl)-5-(non-8-enyl)-pyrrolidine and total CHC is total cuticular hydrocarbons. Total CHC and total alkaloid are represented in µg/500 µL.

The relative recovered amounts of the waxes are visualized in the heat map in Figure 2, except for cholesterol, which was excluded due to its singular profile and small recovered quantity. The hydrocarbons were further divided into compound classes, listed in decreasing order by their recovery in the extract: monomethylalkanes (MeC_n_), alkanes (C_n_), alkenes (C_n_:1), dimethylalkanes (diMeC_n_), alkadienes (C_n_:2) and trimethylalkanes (triMeC_n_) (Table 2). Nelson already stated that methylated alkanes are often found to be the major component in an insect’s epicuticular wax layer [39], which is also the case for the pharaoh ant.

In some cases, multiple compound classes, mostly alkenes and alkadienes, were identified in an overlapped GC peak. All of the identified compounds and isomers are listed in Table 1. In the data presentation of Figure 2 and in Table 2, the compound with the highest intensity of its molecular ion of an overlapped GC peak accounts for the full area of the peak. The full dataset from which Figure 2 is constructed, is provided in Appendix B (Table A1).

From the overview of the recovered epicuticular compounds (Figure 2 and Table 1), it is clear that, overall, hydrocarbon compounds with an uneven carbon number are present in larger quantities than the compounds with an even carbon number. According to Blomquist, this is a consequence of the formation of these components occurring mainly from two carbon units and a subsequent decarboxylation. However, an occasional chain initiation by propionyl-CoA instead of acetyl-CoA can lead to a small quantity of even n-alkanes [26]. Unsaturated hydrocarbons with an even carbon number, with exception of triacontene (x-C_30_:1), were not observed in the wax extracts. In addition, methylated alkanes with an uneven carbon number in the backbone only have isomers with the methyl groups present in uneven positions. For backbones with an even carbon number, methyl branches were located in both even and uneven positions. While the synthesis pathways of terminal alkanes (2-, 3-, 4-, 5- and 6-methylalkanes) are slightly different from the synthesis pathways of internally branched alkanes (7- to center-chain methylalkanes), the difference between methyl positions on even and odd backbones is identical in both cases and also a result of their biosynthetic origin, confirming these findings [26]. The same holds for the dimethyl- and trimethylalkanes which all contained, with the exception of 12,16-dimethyldotriacontane (12,16-diMeC_32_), uneven backbones. The methyl groups on the uneven backbones were always located in uneven positions. The overall composition of the wax layer of the pharaoh ant bears resemblance to the composition presented for the red harvester ant *Pogonomyrmex barbatus* [17]. The main differences lie in the majority compounds, which are n-heptacosane (n-C27) and n-nonacosane (n-C29) in the pharaoh ant extracts and n-pentacosane (n-C25) for the harvester ants [17]. In addition, for the pharaoh ant extracts, the dimethylalkanes and trimethylalkanes consistently show the same methyl group positions, with the second (and third) methyl group being separated by three or seven methylene groups, e.g. 9,13,21-trimethyltritriacontane (9,13,21-triMeC_33_) and 11,15,23-trimethyltritriacontane (11,15,23-triMeC_33_). These distributions are also observed very often in epicuticular wax compositions [26].

### 2.2. Adsorption of Synthetic Alkane Mixture on Zeolites 

To assess the selectivity of adsorption and the efficiency of the extraction procedure using n-hexane, a synthetic alkane mixture (alkane_mix) was prepared containing the two most abundant n-alkanes present in the wax layer of the pharaoh ant, viz. n-heptacosane and n-nonacosane, as well as a methylated compound, 3-methylpentacosane (3-MeC_25_). This product was produced in-house and contained three side-products: 3-methyltetracosane (3-MeC_24_), 3-methylhexacosane (3-MeC_26_) and 3-methylheptacosane (3-MeC_27_). The results of the GC-MS analysis of this alkane mixture and the extracts of the samples loaded with this mixture are provided in Table 3. In Table 3, the compounds are grouped per material class, n-alkanes and monomethylalkanes. Table A2 in Appendix B provides a full account of the data, listed per individual compound.

**Figure 3:**
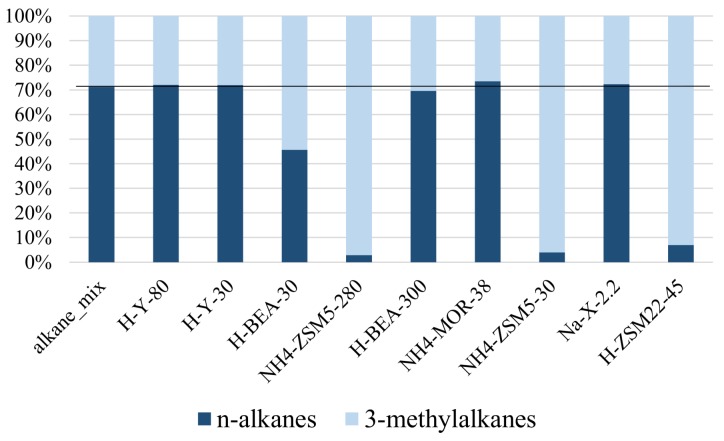
Distribution of n-alkanes and 3-methylalkanes in the synthetic alkane mixture and the extracts of the zeolites loaded with this solution. The n-alkanes are n-C_27_ and n-C_29_, while the 3-methylalkanes are 3-methylpentacosane and its side-products 3-methyl (C_24_, C_26_ and C_27_) with 3-methylpentacosane as main component. The black line shows the distribution in the reference sample (alkane_mix).

These results showed that the analysis of the adsorbed compounds is not trivial. The quantity of hydrocarbons recovered in the n-hexane extracts was systematically lower than the quantity loaded onto the zeolites, indicating that part of the adsorbed hydrocarbons could not be extracted again using n-hexane. The distribution of n-alkanes and monomethylalkanes in the extracts (Figure 3 and Table 3) showed that zeolites H-Y-30, H-Y-80, H-BEA-300, NH4-MOR-38 and Na-X-2.2 are non-selective while other zeolite materials—H-BEA-30, NH_4_-ZSM5-30, NH_4_-ZSM5-280 and H-ZSM22-45—strongly adsorb n-alkanes rather than methylated compounds and thus the extract is enriched with more weakly adsorbed methylated compounds. This effect is less severe in the H-BEA-30 sample, where the percentage of n-alkanes in the extract is still 45.5% compared to 71.1% in total (extractable and non-extractable compounds). In NH_4_-ZSM5-30, NH_4_-ZSM5-280 and H-ZSM22-45, the zeolites with the narrowest pores of the investigated zeolite collection, the amount of n-alkanes in the extract did not even reach 0.5%, indicating these compounds are particularly strongly adsorbed.

These findings on heavy alkanes are in line with earlier reported selectivity patterns for lighter alkanes by Denayer et al. [24]. In that study on NH_4_-ZSM22-91 zeolite, n-alkanes were found to penetrate deeply inside the pores and were adsorbed strongly, while branched alkanes adsorbed on the surface of the zeolite crystals and in pore mouths where the interaction with the zeolite is weaker [24]. Short branched alkanes interact with one pore mouth while longer branched alkanes with (C_12_+) can penetrate in two or more neighboring pore mouths with part of their chains, a mechanism called key-lock adsorption [23,40]. For the small hydrocarbons used by Denayer et al., zeolite Na-Y-60 showed no selectivity towards adsorbing n-alkanes or isoalkanes in its micropores. Zeolite H-ZSM5-274 performed similarly to zeolites mordenite and beta, showing a slight selectivity towards adsorbing n-alkanes over branched compounds in its micropores [24]. For the long hydrocarbon chains present in the epicuticular wax layer and adsorbed onto the zeolites, the ZSM-5 samples used here (NH_4_-ZSM5-30 and NH_4_-ZSM5-280) all strongly adsorbed n-alkanes and weakly adsorbed branched compounds, leading to higher recovery of methylated compounds in the extracts. The same behavior was observed by H-ZSM22-45 (Figure 3 and Table 4). H-BEA-30 also showed selective adsorption of the n-alkanes over the monomethyl-branched compounds, but this selectivity was less pronounced and zeolites H-Y-30, Na-X2.2, NH_4_-MOR-38 and H-BEA-300 did not show any selectivity.

**Figure 4:**
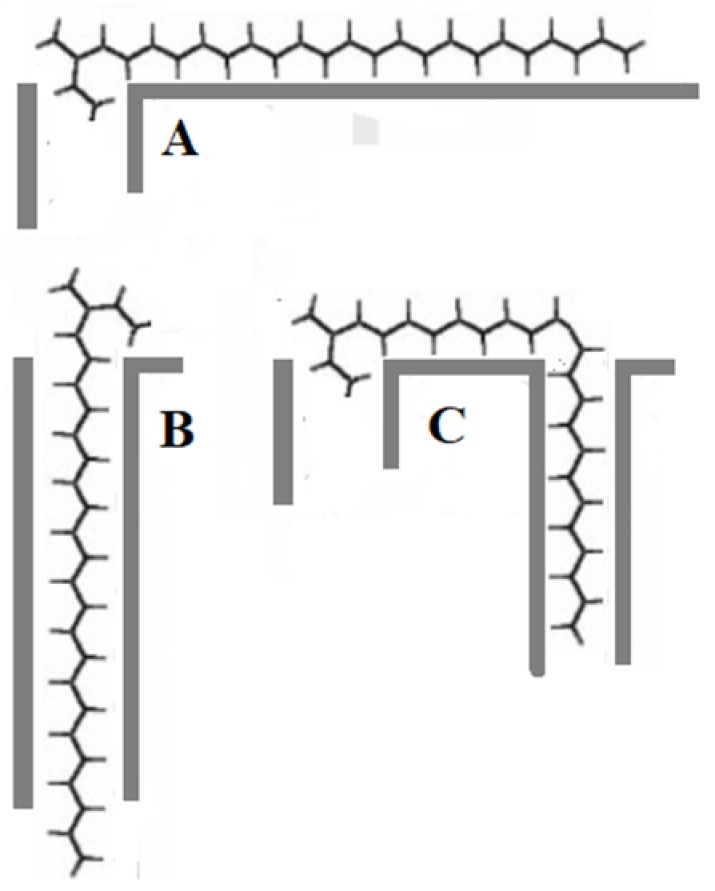
Possible key-lock adsorption configurations of 3-methylpentacosane. (**A)** The configuration with the shortest end of the backbone, before the methyl branch, adsorbed in the pore. The longest end of the backbone is adsorbed on the surface of the material; (**B**) the configuration where the longest end of the backbone is adsorbed in the pore and the shortest end of the backbone is adsorbed on the surface of the material; (**C**) the configuration where the shortest end of the backbone is adsorbed inside a pore and the longest end of the backbone reaches the next pore opening, allowing it to be partially adsorbed inside this pore.

This adsorption selectivity can be explained by the pore sizes of the zeolites (Table 5) which are in decreasing order: Y = X > MOR > BEA > ZSM5 > ZSM22. Overall, as the hydrocarbons get larger, there is a large increase in adsorption enthalpy, but also a large loss of entropy for adsorption in materials with narrow pores. The loss of entropy is caused by immobilizing the hydrocarbon chain inside the pores. This indicates that longer alkanes, when they are adsorbed, are usually very strongly adsorbed in materials with narrow pores. As the n-alkanes become larger, the pore size they can be strongly adsorbed in also appears to become larger. Not only do NH_4_-ZSM5-30 and NH_4_-ZSM5-280 (pore size 0.54 × 0.56 nm) strongly adsorb the n-alkanes, even H-BEA-30 (pore size 0.57 × 0.75 nm) shows selective adsorption of the n-alkanes. Overall, H-BEA-30, NH_4_-ZSM5-30, NH_4_-ZSM5-280 and H-ZSM22-45 strongly adsorb more of all of the compounds in the synthetic alkane mixture, both the n-alkanes and 3-methylalkanes than the other samples, because both can enter the small micropores, at least for the most part, up to the position of the methyl branch [23]. However, it is likely that the 3-methyl branched compounds undergo key-lock adsorption and some of the molecules are adsorbed mostly on the surface of the zeolite, where they are less strongly adsorbed (Figure 4).

The selectivity for H-BEA-300 was different from H-BEA-30, although these samples have the same pore architecture. A possible explanation is that the H-BEA-300 sample has more framework defects, widening the pores, since the high Si/Al ratio of 300 was obtained by a dealumination treatment which damages the zeolite crystals [41]. This assumption is backed by the smaller microporous surface area and larger BET (Brunauer-Emmett-Teller) specific surface area in H-BEA-300 compared to H-BEA-30, indicating the presence of more pores with a larger pore size in H-BEA-300 than H-BEA-30 (Table 6) [21].

### 2.3. Adsorption of Epicuticular Waxes on Zeolites 

The zeolites adsorbed a significant amount of hydrocarbons upon contact with the pharaoh ant foragers. Zeolite Na-X-2.2 was the only material adsorbing some alkaloid components (Table 2) in addition to hydrocarbons. This is probably due to the polarity of this zeolite, since it contains a large amount of sodium cations in its pores. In some zeolites, overlapping peaks of alkenes and alkadienes showed a higher abundance of alkenes, whereas, in the hexane wash of the foragers, the alkadienes were more prominent. In addition, the peak corresponding to dotriacontene (x-C_32_:1), found in some zeolite samples, is largely masked in the ant extract by 3-methylhentriacontane (3-MeC_31_). In addition, two compounds were recovered from these zeolite extracts which could not be defined in the reference samples obtained from the foragers: 7- to 13-methylhexacosane (7- to 13-MeC_26_) and both 9,13,21- and 11,15,23-trimethyltritriacontane (9,13,21- and 11,15,23-triMeC_33_) (Figure 2). The observation of these compounds in extracts from zeolites and not in the hexane wash of the foragers shows that these compounds were more effectively collected on the zeolites than in the n-hexane solvent.

**Figure 5:**
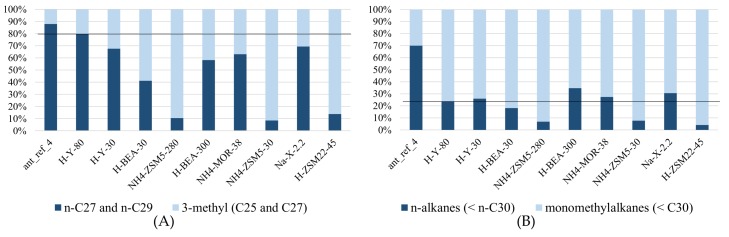
The relative quantities recovered from the n-hexane extracts of the zeolite samples exposed to *M. pharaonis* foragers: (**A**) n-heptacosane (n-C_27_) and n-nonacosane (C_29_) versus 3-methylpentacosane (3-MeC_25_) and 3-methylheptacosane (3-MeC_27_); and (**B**) all n-alkanes smaller than n-triacontane (n-C_30_) versus all monomethylalkanes with backbones smaller than triacontane (<C_30_). For both (**A**,**B**), the distributions of the two groups in the reference sample (H-Y-80) are shown by the black line.

Similar to what was observed with the synthetic alkane mixture, the adsorbed cuticular hydrocarbons could not be quantitatively recovered from the zeolite samples by extraction with n-hexane. The adsorption selectivity was probed by analyzing the uptake of compounds which were also present in the synthetic alkane mixture: n-heptacosane (n-C27), n-nonacosane (n-C29), 3-methylpentacosane (3-MeC25) and 3-methylheptacosane (3-MeC27) (Figure 5A and Table A3). 3-methyltetracosane (3-MeC_24_) and 3-methylhexacosane (3-MeC_26_) were not included, since they were not found in the extracts from the zeolites.

While all zeolite extracts were slightly enriched with 3-methylalkanes, showing that n-alkanes are overall more difficult to extract, the lowest enrichment was present in H-Y-80 sample. This zeolite has the widest pores in the zeolite collection (Table 5) and serves as the reference sample. The extracts from zeolites H-BEA-30, NH4-ZSM5-30, NH4-ZSM5-280 and H-ZSM22-45 were further enriched in 3-methyl branched compounds, showing these zeolites are shape selective. Shape selectivity in alkane adsorption means that the uptake of linear alkanes is preferred over branched ones. Shape selectivity earlier observed with shorter alkanes in the literature was clearly present with epicuticular wax compounds in this research [23,24,40].

**Figure 6:**
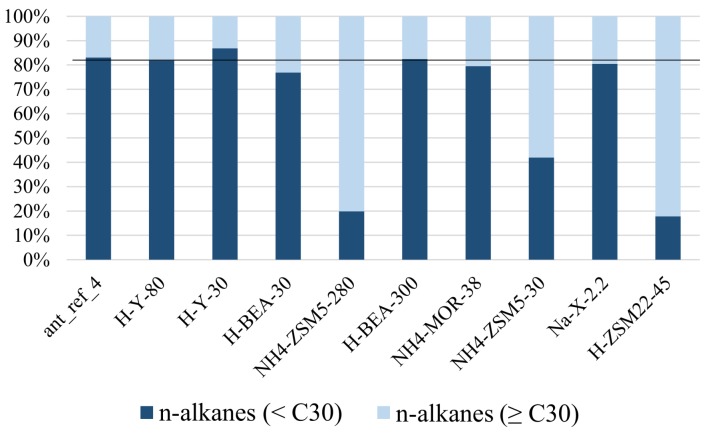
The relative quantities of n-alkanes smaller than n-triacontane (<n-C_30_) versus n-alkanes equal to or larger than triacontane (≥n-C_30_) recovered from the n-hexane extracts of the zeolite samples exposed to *M. pharaonis* foragers. The distributions of the two groups in the reference sample (H-Y-80) are shown by the black line.

Since the epicuticular wax layer of the pharaoh ants consists of more compounds than were present in the synthetic alkane mixture, deeper investigation into the adsorption selectivity of the zeolites was possible. Shape selective adsorption of n-alkanes on zeolites H-BEA-30, NH_4_-ZSM5-30, NH_4_-ZSM5-280 and H-ZSM22-45 is observed for very long chains up to n-C_30_ (n-triacontane) and monomethyl-branched isomers (Figure 5B, Table A3). This adsorptive discrimination among linear and mono-branched alkanes vanished, however, for alkanes containing 30 or more carbon atoms (Figure 6).

The absence of shape selectivity with the longest chains suggests that these very heavy alkanes do not penetrate entirely inside the small pores of zeolites ZSM5, ZSM22 and H-BEA-30. If the increase in adsorption enthalpy does not compensate the loss of entropy, the adsorption of the whole chain would be thermodynamically unfavorable. In this instance, part of the hydrocarbon chain containing the methyl branching would stay outside of the pores as would a similar-sized part of a corresponding n-alkane chain. This leads to a similar Gibbs free energy of adsorption in both cases and thus the absence of selectivity.

**Figure 7:**
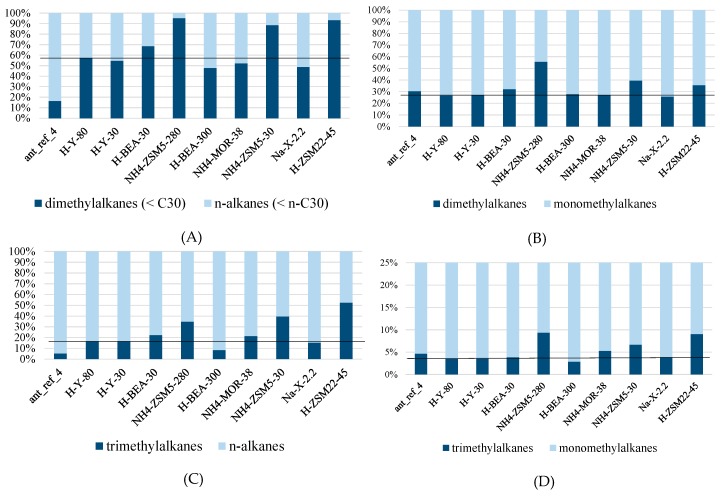
The relative quantities recovered from the n-hexane extracts of the zeolite samples exposed to *M. pharaonis* foragers: (**A**) Dimethylalkanes with backbones smaller than triacontane (<C_30_) versus n-alkanes smaller than n-triacontane (<n-C_30_); (**B**) dimethylalkanes versus monomethylalkanes; (**C**) trimethylalkanes versus n-alkanes; (and **D**) trimethylalkanes versus monomethylalkanes. For (**A**–**D**), the distributions of the two groups in the reference sample (H-Y-80) are shown by the black line.

The dataset was also used to evaluate whether there was adsorption selectivity based on chain length. For this purpose, the n-alkanes were divided in two groups: n-alkanes shorter than C_30_ and C_30_ and longer. This revealed that some zeolites (NH_4_-ZSM5-30, NH_4_-ZSM5-280, H-ZSM22-45, H-BEA-30, Na-X-2.2 and NH_4_-MOR-38) selectively adsorb n-alkanes smaller than n-triacontane (n-C_30_). The ZSM-5 and ZSM-22 zeolites haves the narrowest pores among the investigated zeolites. It is now observed that these zeolites preferentially adsorb shorter chains. These shape selective zeolites, ZSM5 and ZSM22, also showed adsorption preference for n-alkanes over dimethyl- and trimethylalkanes as well as for monomethylalkanes over dimethylalkanes and trimethylalkanes (Figure 7A–D and Table A3).

This is to be expected since branching widens the molecules. Zeolite H-BEA-30 having somewhat wider pores adsorbs n-alkanes preferentially and also discriminates according to chain length, but the selective adsorption is less pronounced in both cases (Figure 5 and Figure 6). Zeolites Na-X-2.2 and NH_4_-MOR-38 do not discriminate according to branching, but do according to chain length. This is surprising, since these zeolites have among the widest pores (Table 7). Zeolite H-BEA-300 was again different because this one shows a preference for adsorbing branched compounds over linear ones. Such a selectivity was coined inverse shape selectivity and may be explained by the tighter fitting of branched isomers in the pores of the zeolite [42].

**Figure 8:**
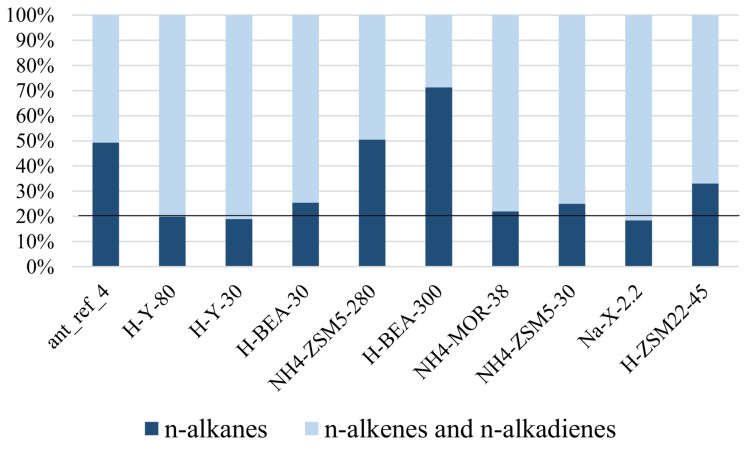
The relative quantities of n-alkanes versus n-alkenes and n-alkadienes recovered from the n-hexane extracts of the zeolite samples exposed to *M. pharaonis* foragers. The distributions of the two groups in the reference sample (H-Y-80) are shown by the black line.

Finally, the data also provided insight on the adsorption selectivity of unsaturated hydrocarbons (Figure 8 and Table A3). Here, the preferential uptake of alkenes and alkadienes is most pronounced on NH_4_-ZSM5-280 and H-BEA-300 (Figure 7A–D and Table A3). There is no straightforward explanation why these zeolites would stand out. NH_4_-ZSM5-30 and H-ZSM22-45 also selectively adsorbed the n-alkenes and n-alkadienes, but the selectivity was less prominent in these samples. This selectivity is also evident in the identification of the epicuticular wax compounds, where dotriacontene (x-C32) was only identified in the extract from H-Y-30, H-Y80, Na-X-2.2 and NH_4_-MOR-38, but, at the same retention time, 3-methylhentriacontane was identified in the extracts from H-BEA-300 and H-ZSM22-45. O’Connor already showed a similar use of preferential adsorption by applying a molecular sieve (5A) to adsorb alkanes from a synthetic mixture to reveal small dimethylalkane peaks which would otherwise be unidentifiable [44].

With exception of the extracts from the more polar zeolite Na-X-2.2, zeolite H-ZSM22-45 and zeolite NH_4_-MOR-38—the three least insecticidal materials—all of the zeolite extracts show an enrichment of alkene over alkadiene components when compared to the hexane wash (Table 2). A full overview of the selectivity types found in the zeolites and their pore sizes is provided in Table 5.

A peculiar note is that NH_4_-MOR-38 shows by far the highest total adsorbed amounts of cuticular hydrocarbons while it was one of the less effective insecticidal materials from previous research [21] (Table 1 and Table 2, and Figure 2). These observations lead us to think not only the adsorption capacity itself is important, but that also the selective adsorption by the insecticidal material of certain components can strongly influence their effectiveness.

## 3. Materials and Methods

### 3.1. Pharaoh Ant Colonies

The first colony of pharaoh ants was obtained from Purdue University, Indiana, USA. This colony was then further reared in a climate room and split up into different trays, obtaining several (sub)colonies. The climate room was kept at 27 °C and 70% relative humidity at all times. The nesting spaces provided were opaque petri dishes, where the bottom was covered with a layer of moisturized plaster. The colonies were provided with a constant supply of fresh water and were fed weekly with sugar water and protein rich food such as grasshoppers, mealworms, egg yolk and cat food.

### 3.2. Adsorption Materials: Zeolites

The materials used for the adsorption experiments were zeolite powders, used in previous research to determine their insecticidal effectiveness [21]. The median survival times (LT_50_) and microporous surface areas obtained from that research are mentioned in Table 6 and the zeolite structures along with the most important parameters defining these structures are also provided in Table 7. With the exception of H-ZSM22-57, which was made in-house, all of the zeolite materials were obtained from a commercial source.

### 3.3. Epicuticular Wax Analysis

#### 3.3.1. Sample Preparation for GC-MS Analysis of Epicuticular Hydrocarbons and Adsorption by Zeolite Materials

For the reference samples, 120, 300, 1200 or 1200 foragers were collected from the colony, immersed in 1.5 mL of n-hexane and vortexed for 0.5, 1, 2 and 5 min, respectively, to extract the epicuticular compounds (Table 8).

A similar method was used by Castner and Nation in 1986 [20], who immersed mole crickets in pentane and slightly agitated them for 1 min, and Lok B. and Nelson D., who used hexane and pentane, respectively, to immerse ants for a total of 10 min with no mention of stirring [17,18]. We opted to use n-hexane (Acros organics, HPLC grade, 95%) for immersion and used different amounts of foragers and various vortex times to assess the differences in the results. The solvent was then evaporated completely, leaving a sample residue in the GC-MS vial. Before measuring, 500 µL of fresh n-hexane was added to the vial and vortexed for 1 min, after which the solution was injected in the GC-MS instrument.

Wax component adsorption on the zeolite powders was investigated as follows. For every sample, 15 cylindrical jars, with a diameter of 3 cm and a height of 7 cm, were filled with 50 mg of zeolite powder and 120 foragers (or no foragers for the blanks). The jars were internally coated with a commercially obtained Fluon solution (polytetrafluorethene) to prevent the ants from escaping. After 1 h of exposure, the ants were removed from the jars and the powders were combined. Since some of the powder was removed together with the ants, an exact amount of 500 mg from the collected zeolite powder was added to a glass vial. All of the samples were produced in triplicate, including the blanks. Every sample was then extracted using 3 mL n-hexane. The mixture was sonicated for 30 min and subsequently the zeolite particles were separated by centrifugation at 3200 rpm for 15 min. Then, 1.5 mL of n-hexane extract was pipetted into a GC vial and left open to evaporate the n-hexane. The zeolite powder left in the glass vial was also left to dry. The wax molecules from the epicuticle are long alkanes, which are expected to be non-volatile and therefore they were assumed to be left as a residue. The dried zeolite powder was then extracted a second time with n-hexane to recover the remaining adsorbed compounds. The second extract was pipetted into the same GC-vial as the first extract and evaporated. Before measuring, 500 µL of fresh n-hexane was added to the GC vials, after which they were vortexed for 1 min and analyzed with GC-MS.

For evaluating the efficiency of the extraction of hydrocarbons from the zeolite powders using n-hexane, a synthetic mixture of reference alkanes was used (Table 8). For this purpose a synthetic solution in n-hexane was prepared with equal amounts (1.05 mg) of (*R*)-3-methylpentacosane (synthesized in-house), n-heptacosane (Fluka, >99.5%) and n-nonacosane (Fluka, >99.5%), with n-heptacosane and n-nonacosane being the two alkanes recovered in largest quantities from the ant references and n-alkanes and monomethylalkanes being the most prominent component classes found in the epicuticular wax (Table 2). The solution was then diluted to a total volume of 25 mL. From this solution, 0.5 mL was loaded onto 500 mg of zeolite powder. Then, 1.5 mL of n-hexane was added and the mixture was sonicated for 30 min, after which the sample vial was left open to evaporate the n-hexane. The two extraction procedures were performed on the zeolite powder in the same vial as the loading with the synthetic mixture and according to the description in the previous section. Performing the extraction procedure in the same vial was beneficial to the quantification since all of the compound added is either adsorbed on the zeolite or left unadsorbed in the vial. The amount of synthetic compounds which were strongly adsorbed onto the zeolite and thus not recovered in the extract could then be determined easily from the total amount of synthetic solution added. All of the loaded samples were prepared in duplicate.

While activated carbon showed a higher insecticidal effectivity than zeolites in previous research [21,45], extractions with n-hexane were only successful on zeolites, and therefore the study was performed using zeolites only.

#### 3.3.2. GC-MS Analysis

GC-MS analysis was performed on a Thermo Trace 1300 gas chromatograph, using a Thermo Scientific ISQ mass spectrometer with electron ionization source (70 eV). The system was equipped with a 30 m long Restek MXT-5 column with a diameter of 0.25 mm coated with a stationary phase, consisting of 5% diphenyl and 95% dimethylpolysiloxane and a thickness of 0.25 µm. A split injection was used with a 1/3 split ratio, an injection volume of 1 μL, an inlet temperature of 320 °C and a helium carrier gas flow of 0.9 mL/min. The column temperature was programmed as follows: 2 min at 40 °C, increase to 120 °C at 20 °C/min, increase to 200 °C at 10 °C/min, increase to 250 °C at 7 °C/min, increase to 350 °C at 5 °C/min, 4 min hold at 350 °C. The transfer line and the ion source were kept at 300 °C, the solvent delay time was set at 3 min 40 s, the examined mass range of the scans was 33–720 amu and the scan time was 0.304 s. A reference linear C_7_ to C_40_ linear alkane ladder standard (49452-U, Supelco (Bellefonte, PA, USA) at three different concentrations (0.001, 0.01 and 0.1 µg/mL) was run to determine retention times and retention indices, and for determining response factors. 

#### 3.3.3. GC-MS Data Processing 

The peaks of the total ion chromatograms were defined and integrated using an in-house developed R-script [46] (available from the authors on request) and retention indices were calculated using cubic-spline interpolation based on the elution times of the aforementioned alkane ladders [47]. After verification the relationship between peak area and concentration was linear on a log-log scale [46], quantification of all hydrocarbon compounds was performed using interpolation on a log-log scale, using the peak areas of the closest eluting n-alkane of the external alkane ladders for each compound.

The resulting values were averaged over all the replicates per forager sample, loaded sample or blank and compounds recovered from the blank were then subtracted from the measured forager sample and loaded sample amounts. Cuticular compounds in the samples were identified based on their mass spectra and retention indices [46], and via comparison with other studies, standards and published spectra [31,33,38,48]. The exact positions of alkene and alkadiene double bonds could not be determined.

#### 3.3.4. Assessing Selective Adsorption

Statistical analysis was performed on the n-hexane extracts to detect significant selectivity differences of hydrocarbon adsorption on the different zeolites. To perform post hoc tests on proportional data to assess whether the zeolites selectively adsorbed certain compounds, we first transformed the data using the so-called Aitchison or centered log-ratio transformation. This was done in R version 3.2.3. by applying the function clr from the package compositions. We then used the functions aov and lsmeans from the packages stats and lsmeans, respectively, to perform post hoc treatment versus control testing of the zeolite extracts compared to the reference. As a reference for the loaded samples, the synthetic alkane mixture (alkane_mix) was used and performed very well. For the n-hexane extracts from the zeolites exposed to the foragers, the analysis was more difficult, since the n-hexane wash from the ants (ant_ref_4) was not always representative as a reference due to the much shorter contact time with the ants (5 min as opposed to 1 h for the zeolites). Zeolite H-Y-80 was then picked as the reference sample, since this is the material with the largest pore size (Table 7) and between H-Y-30 and H-Y-80, the latter has the highest BET (Brunauer–Emmett–Teller) specific surface area and the smallest microporous surface area, indicating it has a higher proportion of larger pores and is assumed to be the least selective upon adsorption of compounds (Table 6). This assumption is also backed up by its proven absence of selectivity when loaded with the synthetic alkane mixture (Table 3). Statistical tests resulting in a *p*-value < 0.05 were considered significant.

## 4. Conclusions

This manuscript communicates the complete composition of the epicuticular wax layer of the pharaoh ant, obtained using multiple hexane washes of the foragers. This revealed 53 peaks each corresponding to one or more alkaloids or compounds in the wax layer. Of these compounds, four were not previously identified in *Monomorium* species: α-farnesene, cholesterol, 2-pentyl-5-(1-hex-5-enyl)-1-pyrroline and 2-(1-hexenyl)-5-(hept-6-enyl)-1-pyrroline. We also showed the increased benefit of a longer contact time with n-hexane to obtain a better resolution of the peaks, considering that saturation of the column with alkaloid compounds should not occur.

To obtain a more complete profile of the epicuticular wax layer of the pharaoh ant, we also extracted the epicuticular waxes using different types of zeolites, each with their own selectivity in adsorbing the compounds from the epicuticular wax layer of the pharaoh ant. Using zeolites to adsorb the epicuticular wax compounds also resolves the problem of saturating the column with alkaloids when using a too highly concentrated hexane wash, since zeolites do not adsorb the alkaloid components (with exception of the very polar zeolite Na-X-2.2). This analysis using zeolites resulted in the identification of three extra compounds: dotriacontene, 7- to 13-methylhexacosane and both 9,13,21- and 11,15,23-trimethyltritriacontane. While the identification of the last two compounds were most likely a result of the extended contact time with the zeolites—1 h as opposed to 5 min for the hexane wash—the identification of dotriacontene appears to be the result of selective adsorption of 3-methylhentriacontane onto the zeolites.

To obtain a measure of the selectivity of these zeolites and the efficiency of the hexane extractions, we also performed extractions after loading the zeolites with a synthetic alkane mixture (alkane_mix). This synthetic mixture contained the most abundant n-alkanes from the epicuticular wax layer and a 3-methyl branched compound. The analysis, showing preferential adsorption of the linear compounds on some zeolites, provided us with valuable insight into the adsorption mechanisms that are present when exposing powdered zeolite materials to these types of long hydrocarbon chains, such as the importance of the key-lock adsorption mechanism.

The analysis of the adsorption of the cuticular waxes onto the zeolites was further useful in analyzing the selective adsorption on zeolites of long hydrocarbon compounds. It not only allowed us to replicate the same selectivity profiles, it also allowed us to define the boundaries of the selectivity with respect to hydrocarbon chain length, since the selectivity was no longer present when comparing compounds larger than n-triacontane (n-C_30_) and 3-methyltriacontane (3-MeC_30_), respectively. This enticed us to compare selective adsorption of smaller n-alkanes (<n-C30) versus larger n-alkanes (≥n-C30) on the zeolites, revealing that the shape selective zeolites (H-ZSM22-45, NH_4_-ZSM5-30, NH_4_-ZSM5-280 and H-BEA-30) as well as zeolite Na-X-2.2 and NH_4_-MOR-38 selectively adsorbed components smaller than n-triacontane (n-C30). We found that, in general, the shape selective zeolites ZSM-22 and ZSM-5 showed the most pronounced selectivity for shorter compounds and compounds with the least branching, which can be easily explained through their small pore size. H-BEA-300 on the other hand showed inverse shape selectivity, selectively adsorbing more bulky, branched compounds. Finally, we also obtained information regarding the selectivity of NH_4_-ZSM5-280 and H-BEA-300 to strongly adsorb n-alkenes and n-alkadienes. This kind of information can be of great interest, not only for the field of entomology, but also for the fields of catalysis and refinery, where often very long hydrocarbons are used in production processes. Information about the selective adsorption of different compounds could be crucial in obtaining a selective product from a reaction or cracking process [23].

These observations show (both size and shape) selectivity for many of the zeolite samples used in the study. These zeolites also showed a wide variety of mean survival times in previous research and no straightforward link to the adsorbed quantity of the epicuticular wax compounds could be determined, which suggests that selectivity plays a major role in their insecticidal effectiveness. In addition, since the highest quantities of epicuticular wax compounds were recovered from zeolite NH_4_-MOR-38 extracts and this zeolite showed little insecticidal activity in previous research—the first forager died after 1.5 h [21]—wax extraction directly from the foragers or other insects using this zeolite can prove to be a good non-lethal extraction method. However, more research in this area is advised.

## Figures and Tables

**Table 1:** List of components identified in the GC-MS spectra represented in Figure 2. Numbers refer to the peak numbering in Figure 2. Indices are used to indicate in which samples the components were identified: * present in all samples, ** present in all samples except Ant_ref_1, *** present in both Ant_ref_3 and Ant_ref_4 and finally **** only present in Ant_ref_4. Components presented as C_x_ are components which were not attributed to *Monomorium* species before; they were not included in Figure 2. Representation of the components is as follows: Alkanes (C_n_), alkenes (C_n_:1), dienes (C_n_:2), monomethylalkanes (MeC_n_), dimethylalkanes (diMeC_n_), and trimethylalkanes (triMeC_n_).

List of Components Present in the Hexane Wash of *M. pharaonis*					
1) monomorine I	*	16) 3-MeC25	***	34) 8- to 15-MeC30	*
2) monomorine II	*	17) n-C26	*	35) 12,16-diMeC30	***
C_1_) α –farnesene	*	18) x,y-C27:2 and x-C27:1	*	36) x-C31:1 and x,y-C31:2	*
C_2_) 2-pentyl-5-(hex-5-enyl)-1-pyrroline	*	19) n-C27	*	37) n-C31	*
3) monomorine III	*	20) 7-, 9-, 11-, 13-MeC27	*	38) 7-, 9-, 11-, 13-, 15-MeC31	*
C_3_) 2-(1-hexenyl)-5-(hept-6-enyl)-1-pyrroline	*	21) 5-MeC27	***	C4) cholesterol	*
4) monomorine IV	*	22) 11,15-diMeC27	*	39) 11,15-diMeC31	*
5) x-C19:1	**	23) 3-MeC27	*	40) 3-MeC31 and x-C32	**
6) n-C19	****	24) n-C28	*	41) n-C32	***
7) n-C20	***	25) 9- to 14-MeC28	*	42) 3,7,11-triMeC31	**
8) *trans*-2-(1-hex-5-enyl)-5-(non-8-enyl)-pyrrolidine	*	26) x-,y-C29:2 and x-C29:1	*	43) x,y-C33:2	***
9) n-C21	****	27) y-C29:1	**	44) x-C33:1	***
10) n-C22	***	28) n-C29	*	45) y-C33:1	*
11) n-C23	*	29) 4,8,12-triMeC28	***	46) 17-, 15-, 13-, 11-MeC33	**
12) n-C24	*	30) 7-, 9-, 11-, 13-, 15-MeC29	*	47) 11,15- and 9,13-diMeC33	*
13) x-C25:1	**	31) 11,15-diMeC29	*	48) 11-, 13-, 15-MeC35	***
14) n-C25	*	32) 3-MeC29 and x-C30:1	*	49) 11,15- and 13,17-diMeC35	***
15) 9-, 11-, 13-MeC25	*	33) n-C30	*		

**Table 2:** Distribution (µg/500 µL and %) of the CHC (cuticular hydrocarbons, without alkaloids) present in the hexane extract of *M. pharaonis* and samples extracted from zeolites exposed to *M. pharaonis* foragers for 1 h. Components are grouped by class. Total CHC, total alkaloids and total compounds (both CHC and alkaloids) are also provided. Compound classes which were not represented were assigned a value of 0.001 µg (roughly 10% of the detection limit of the analysis).

Compound/Sample	Ant_ref_4		HY80		HY30		HBEA30		NH4ZSM5280		HBEA300		NH4MOR38		NH4ZSM530		NaX2.2		HZSM2245	
	µg	%	µg	%	µg	%	µg	%	µg	%	µg	%	µg	%	µg	%	µg	%	µg	%
alkane	13.134	26.9	4.249	7.9	2.530	7.7	1.175	6.8	1.048	7.0	2.692	17.1	8.903	8.7	1.050	5.0	4.033	8.6	2.053	4.7
alkene	11.288	23.1	17.045	31.8	10.883	33.1	3.441	19.8	1.027	6.9	1.083	6.9	27.345	26.6	3.072	14.7	15.037	32.1	3.535	8.1
alkadiene	2.197	4.5	0.001	0.0	0.001	0.0	0.001	0.0	0.001	0.0	0.001	0.0	4.377	4.3	0.082	0.4	2.900	6.2	0.624	1.4
monomethylalkane	14.991	30.7	23.009	43.0	13.822	42.0	8.440	48.5	5.452	36.5	8.475	53.7	43.628	42.4	9.655	46.2	17.915	38.3	22.800	51.9
dimethylalkane	6.538	13.4	8.393	15.7	5.131	15.6	4.010	23.0	6.839	45.8	3.273	20.7	16.191	15.7	6.321	30.3	6.225	13.3	12.638	28.8
trimethylalkane	0.733	1.5	0.833	1.6	0.508	1.5	0.339	2.0	0.563	3.8	0.251	1.6	2.445	2.4	0.692	3.3	0.725	1.6	2.261	5.1
total CHC	48.881	5.4	53.529	100	32.874	100	17.405	100	14.929	100	15.774	100	102.889	100	20.87	100	46.835	57	43.911	100
total alkaloids	860.816	94.6	0.001	0	0.001	0	0.001	0	0.001	0	0.001	0	0.001	0	0.001	0	35.311	43	0.001	0
Total compounds	909.797	100	53.530	100	32.875	100	17.406	100	14.930	100	15.775	100	102.890	100	20.871	100	82.146	100	43.912	100

**Table 3:** Distribution (µg/500 µL and %) of the compounds present in the synthetic alkane mixture (alkane_mix) and in the zeolite samples loaded with this solution. Components are grouped as weakly adsorbed (recuperated in the n-hexane extract) and strongly adsorbed (not recuperated in the n-hexane extract) components. These groups are then further divided by class, alkane and monomethylalkane, since these were the only types of compounds present in the alkane mixture.

Compound/Sample	Alkane_Mix		HY80		HY30		HBEA30		NH4ZSM5280		HBEA300		NH4MOR38		NH4ZSM530		NaX2.2		HZSM2245	
	µg	%	µg	%	µg	%	µg	%	µg	%	µg	%	µg	%	µg	%	µg	%	µg	%
weakly adsorbed	60.791	100	27.734	45.6	27.376	45.0	11.126	18.3	2.609	4.3	19.810	32.6	19.800	32.6	2.543	4.2	20.479	33.7	3.846	6.3
alkane	43.222	71.1	19.967	72	19.672	71.8	5.062	45.5	0.074	2.8	13.768	69.5	14.548	73.4	0.101	4.4	14.813	72.3	0.269	6.9
monomethylalkane	17.569	28.9	7.767	28	7.704	28.2	6.064	54.5	2.535	97.1	6.042	30.5	5.252	26.6	2.442	96.0	5.666	27.7	3.577	93.0
strongly adsorbed	0	0	33.057	54.4	33.415	55.0	49.665	81.7	58.182	95.7	40.981	67.4	40.991	67.4	58.248	95.8	40.312	66.3	56.945	93.7
alkane	0	71.1	23.255	70.3	23.550	70.5	38.160	76.8	43.148	74.2	29.454	71.9	28.674	70.0	43.121	74.0	28.409	70.5	42.953	75.4
monomethylalkane	0	28.9	9.802	29.7	9.865	29.5	11.505	23.2	15.034	25.8	11.527	28.1	12.317	30.	15.127	26.0	11.903	29.5	13.992	24.6

**Table 4:** Results of the post hoc tests on the Aitchison transformed data to assess the difference between the proportions of n-alkanes and 3-methylalkanes present in the hexane extracts of the loaded zeolites compared to the reference (synthetic alkane mixture). Due to the presence of only two different compound classes, the results for both classes are identical with the exception of the sign of the estimate, which would be opposite for the 3-methylalkanes. The estimates are given together with their standard errors, *t*- and *p*-values from which the significance can be determined. * indicates a significant difference (*p* < 0.05).

Compound Class	Contrast	Estimate	Standard Error	*t*	*p*	
n-alkanes	HBEA30_load-ref	−0.55	0.05	−10.31	2.77 × 10^−6^	*
n-alkanes	HBEA300_load-ref	−0.05	0.05	−1.00	0.34	
n-alkanes	HY30_load-ref	−0.01	0.05	−0.14	0.89	
n-alkanes	HY80_load-ref	−0.01	0.05	−0.08	0.93	
n-alkanes	HZSM2245_load-ref	−1.79	0.05	−33.45	9.40 × 10^−^^11^	*
n-alkanes	NaX2.2_load-ref	0.01	0.05	0.15	0.88	
n-alkanes	NH4MOR38_load-ref	0.02	0.05	0.45	0.66	
n-alkanes	NH4ZSM5280_load-ref	−2.21	0.05	−41.28	1.43 × 10^−11^	*
n-alkanes	NH4ZSM530_load-ref	−2.26	0.05	−42.12	1.19 × 10^−11^	*

**Table 5:** The different powdered zeolite samples used, their respective theoretical pore size and cage diameters [22] and the observed selectivity types with regards to the cuticular hydrocarbons. When several zeolites showed the same selectivity type, but the extent was very different, the zeolite(s) with the least selectivity are indicated as “little selective”. H-Y-80 is used as a reference and thus not selective by default.

Material	Pore Size (nm^2^) *	Cage Diameter (nm)	Selectivity Type(s)
H-ZSM22-45	0.45 × 0.55	0.571	Shape selective, size selective, little selective adsorption of unsaturated hydrocarbons
Na-X-2.2	0.74 × 0.74	1.124	little size selective
NH_4_-ZSM5-30	0.54 × 0.56	0.636	Shape selective, size selective, little selective adsorption of unsaturated hydrocarbons
NH_4_-MOR-38	0.65 × 0.70	0.670	little size selective
H-BEA-300	0.66 × 0.67	0.668	Inverse shape selective, selective adsorption of unsaturated hydrocarbons
NH_4_-ZSM5-280	0.54 × 0.56	0.636	Shape selective, size selective, selective adsorption of unsaturated hydrocarbons
H-BEA-30	0.66 × 0.67	0.668	Little shape selective, little size selective
H-Y-30	0.74 × 0.74	1.124	None
H-Y-80	0.74 × 0.74	1.124	None

* The pore size mentioned is the theoretical pore size of the zeolite structure. The final resulting pore size can also be influenced by dealumination, which introduces flaws in the framework, widening the pores.

**Table 6:** Zeolite samples, their origin and composition. The zeolite sample notation is structured A-B-X with A the cation exchanged in the zeolite, B the zeolite name and X the silica over alumina (SiO_2_/Al_2_O_3_) ratio of the material. BET is the BET specific surface area, Sp_micro_ is the microporous surface area of the material and LT_50_ is median survival time of *M. pharaonis* foragers exposed to the zeolite, all of which were previously determined [21].

Material	Sample Name	Supplier	Supplier Location	Idealized Chemical Formula	BET (m^2^/g)	Sp_micro_ (m^2^/g)	LT_50_ (min)
H-ZSM22-45	NA	In-house	NA	H_2_(SiO_2_)_57_(Al_2_O_3_)	52	43.1	390
Na-X-2.2	Na-X-2.2	Uetikon	Frankfurt, Germany	Na_22_(SiO_2_)_22_(Al_2_O_3_)_10_	735	2202	330
NH_4_-ZSM5-30	CBV3024E	Zeolyst	Delfzijl, the Netherlands	(NH_4_)_2_(SiO_2_)_30_(Al_2_O_3_)	550	1431	240
NH_4_-MOR-38	CBV30A	Zeolyst	Delfzijl, the Netherlands	(NH_4_)_2_(SiO_2_)_38_(Al_2_O_3_)	613	1249	180
H-BEA-300	CP811C300	Zeolyst	Delfzijl, the Netherlands	H_2_(SiO_2_)_300_(Al_2_O_3_)	781	973	85
NH_4_-ZSM5-280	CBV28014	Zeolyst	Delfzijl, the Netherlands	(NH_4_)_2_(SiO_2_)_280_(Al_2_O_3_)	440	936	85
H-BEA-30	H-BEA-30	Süd-Chemie	München, Germany	H_2_(SiO_2_)_30_(Al_2_O_3_)	624	1028	80
H-Y-30	CBV720	Zeolyst	Delfzijl, the Netherlands	H_2_(SiO_2_)_30_(Al_2_O_3_)	830	861	40
H-Y-80	CBV780	Zeolyst	Delfzijl, the Netherlands	H_2_(SiO_2_)_80_(Al_2_O_3_)	874	793	40

**Table 7:** Structure and framework properties of the different zeolite types. Structure refers to the official framework type of the zeolites, pore size are the dimensions of the largest (accessible) pore in the material [22]. Cage diameter is the diameter of the internal cages, pore direction refers to the amount of dimensions the pores span and Vol_accessible_ refers to the percentage of accessible volume in the zeolite framework (theoretical value based on complete framework) [43].

Material	Structure	Pore Type	Pore Size (nm²)	Cage Diameter (nm)	Pore Direction	Vol_accessible_
H-ZSM22-45	TON	10-ring	0.45 × 0.55	0.571	1D	8.04%
Na-X-2.2	FAU	12-ring	0.74 × 0.74	1.124	3D	27.42%
NH_4_-ZSM5-30	MFI	10-ring	0.54 × 0.56	0.636	3D	9.81%
NH_4_-MOR-38	MOR	10-ring	0.65 × 0.70	0.670	1D*	12.27%
H-BEA-300	BEA	12-ring	0.66 × 0.67	0.668	3D	20.52%
NH_4_-ZSM5-280	MFI	10-ring	0.54 × 0.56	0.636	3D	9.81%
H-BEA-30	BEA	12-ring	0.66 × 0.67	0.668	3D	20.52%
H-Y-30	FAU	12-ring	0.74 × 0.74	1.124	3D	27.42%
H-Y-80	FAU	12-ring	0.74 × 0.74	1.124	3D	27.42%

* The pore direction of MOR is in fact 2D, however the second dimension has a pore size of 0.295 nm (8-ring), which is significantly smaller than the pore size in the main direction and is thus not taken into account.

**Table 8:** List of reference samples and alkane mixture to be loaded onto the zeolites.

Sample	Description
Alkane_mix	Solution of equal amounts (1,05 mg) of 3-MeC25, n-C27 and n-C29 in 25 mL n-C6
Ant_ref_1	Reference sample: 120 *M. pharaonis* foragers vortexed in n-C6 for 0.5 min
Ant_ref_2	Reference sample: 300 *M. pharaonis* foragers vortexed in n-C6 for 1 min
Ant_ref_3	Reference sample: 1200 *M. pharaonis* foragers vortexed in n-C6 for 2 min
Ant_ref_4	Reference sample: 1200 *M. pharaonis* foragers vortexed in n-C6 for 5 min

**Figure A1:**
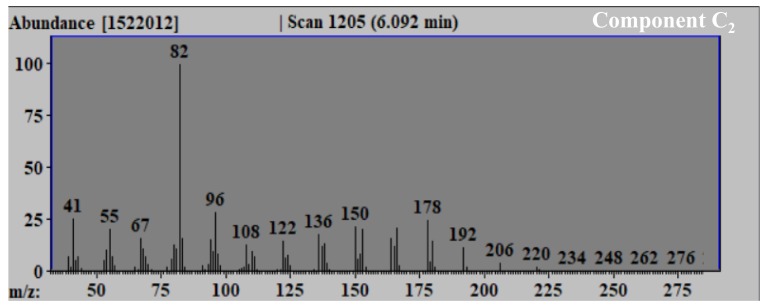
ion spectrum of the C_2_ component in the hexane wash of the foragers (ant_ref_4) obtained by GC-MS analysis.

**Figure A2:**
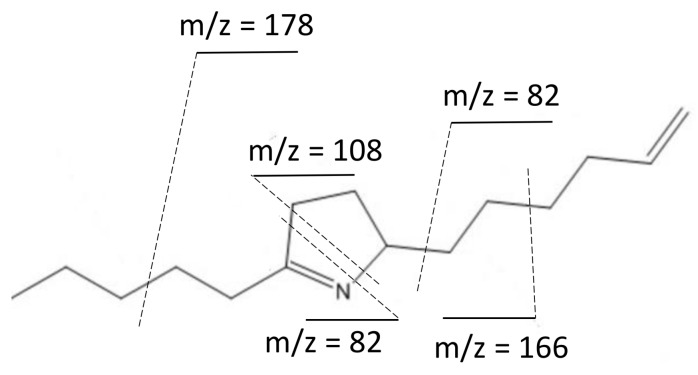
Suggested occurences of cleaving of the C_2_ molecule with the corresponding *m*/*z* of the generated ion.

**Figure A3:**
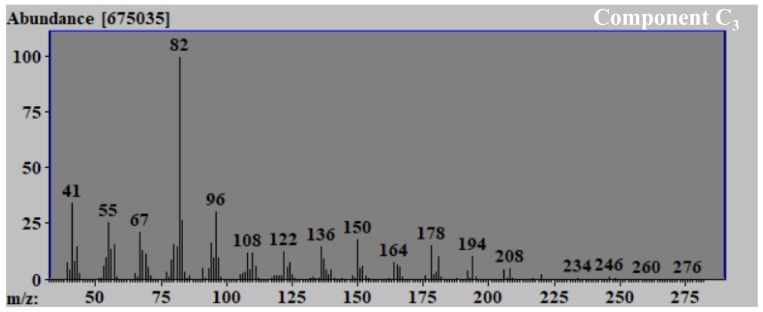
ion spectrum of the C_3_ component in the hexane wash of the foragers (ant_ref_4) obtained by GC-MS analysis.

**Figure A4:**
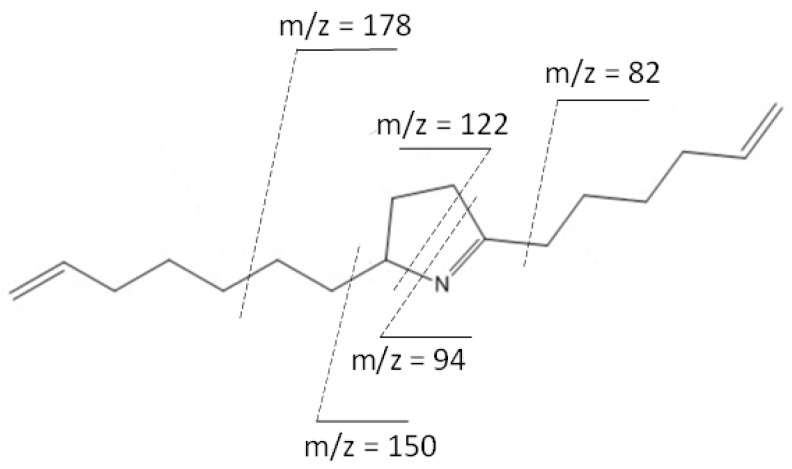
Suggested occurences of cleaving of the C_3_ molecule with the corresponding *m*/*z* of the generated ion.

## Abbreviations

GC	Gas Chromatography
GC-MS	Gas Chromatography—Mass Spectroscopy

## Appendix A

Identification of the pyrroline compounds C_2_ and C_3_

C_2_: 2-pentyl-5-(hex-5-enyl)-1-pyrroline

Mass spectrum obtained by GC-MS analysis

Molecular ion: M^+^ = 220

The presence of both ions at 150/152, 164/166, and 178/180 indicates the presence of both an alkyl and an alkenyl branch.

C_3_: 2-(1-hexenyl)-5-(hept-6-enyl)-1-pyrroline

Mass spectrum obtained by GC-MS analysis

Molecular ion: M^+^ = 246

## Appendix B

Additional tables and figures

**Table A1:** Full dataset of compounds (in µg/500 µL injected sample) obtained by GC-MS analysis for the hexane wash of the foragers (ants_ref_4) and the hexane extracts of the powdered zeolite samples exposed to the *M. pharaonis* foragers. For plotting purposes (Figure 2), compounds which were not recovered (0 µg/500µL) were assigned a value of 0.001 µg/500 µL (roughly 10% of the detection limit).

Compound	Ants_ref_4	HY80	HY30	HBEA30	NH4ZSM5280	HBEA300	NH4MOR38	NH4ZSM530	NaX2.2	HZSM2245
n-C19	0.488	0	0	0.134	0	0	0	0	0	0
n-C20	0.563	0	0	0	0	0.477	0	0	0	0
n-C22	0.224	0	0	0	0	0.211	0	0	0	0.03
n-C23	0.19	0.064	0.057	0.045	0.021	0.026	0.109	0.028	0.077	0.016
n-C24	0.164	0	0	0	0	0.214	0.085	0.058	0.197	0.033
n-C25	1.346	0.932	0.903	0.354	0.055	0.658	2.265	0.116	1.004	0.163
n-C26	0.347	0	0	0.022	0	0.095	0.23	0	0.113	0
n-C27	3.686	1.662	0.83	0.188	0.046	0.347	2.679	0.062	1.169	0.124
n-C28	0.392	0	0	0.054	0.086	0.099	0.421	0.145	0	0
n-C29	3.51	0.817	0.407	0.106	0	0.092	1.29	0.032	0.687	0
n-C30	0.463	0.319	0.163	0.127	0.365	0.25	0.74	0.158	0	0.672
n-C31	1.382	0.455	0.17	0.106	0	0.037	0	0	0.362	0
n-C32	0.379	0	0	0.039	0.475	0.186	1.084	0.451	0.424	1.015
x-C19:1	1.606	0	0	0.174	0	0	0	0	0	0
x-C25:1	0.217	0.144	0.09	0.023	0.103	0	0.167	0.203	0.086	0
x-C27:1	0	1.077	0.636	0.265	0.177	0	1.759	0.359	0	0
x-C29:1	0	2.084	1.357	0.323	0.105	0.021	0	0.335	0	0
y-C29:1	0.357	0.691	0.263	0.121	0	0	0.96	0	0.432	0.182
x-C30:1	0.261	0.529	0.257	0.105	0	0	0	0	0.317	0.312
x-C31:1	7.269	10.567	7.01	2.409	0.642	1.062	19.435	1.995	12.255	3.041
x-C32:1	0	0.566	0.284	0	0	0	0.969	0	0	0
x-C33:1	0.359	0.294	0.294	0	0	0	1.115	0	0.402	0
y-C33:1	1.219	1.093	0.692	0.021	0	0	2.94	0.18	1.545	0
x-,y-C27:2	0.619	0	0	0	0	0	0	0	0.818	0.199
x-,y-C29:2	1.119	0	0	0	0	0	3.754	0	1.794	0.425
x-,y-C33:2	0.459	0	0	0	0	0	0.623	0.08	0.288	0
3-MeC24	0	0	0	0	0	0	0	0	0	0
9-,11-,13-MeC25	0.095	0.214	0.132	0.062	0.091	0.041	0.337	0.102	0.194	0.081
3-MeC25	0.463	0.103	0	0	0.103	0.088	0.353	0.256	0.175	0
7- to 13-MeC26	0	0	0	0.031	0	0	0.289	0.125	0	0.188
3-MeC26	0	0	0	0	0	0	0	0	0	0
7-,9-,11-,13-MeC27	1.062	3.336	1.841	1.047	0.874	1.27	5.216	1.524	1.805	1.827
5-MeC27	0.08	0.176	0.08	0.055	0.033	0.063	0.248	0.042	0.08	0.124
3-MeC27	0.521	0.53	0.592	0.42	0.288	0.227	1.968	0.754	0.643	0.771
9- to 14-MeC28	0.431	0.859	0.347	0.257	0.387	0.139	1.522	0.557	0.542	0.999
7-,9-,11-,13-,15-MeC29	3.094	6.733	3.937	2.674	1.449	2.716	11.375	2.984	4.833	5.408
3-MeC29	0.367	0.394	0.3	0.198	0.154	0.125	0.856	0.274	0.388	0.545
8- to 15-MeC30	0.775	1.379	0.567	0.489	0.903	0.482	2.981	0.954	1.05	2.323
7-,9-,11-,13-,15-MeC31	4.491	6.356	4.014	2.516	0.853	2.495	11.763	1.546	5.439	6.394
3-MeC31	0.725	0	0	0	0	0.132	0	0	0.542	0.581
17-,15-,13-,11-MeC33	2.101	2.227	1.626	0.656	0.317	0.697	5.078	0.537	2.224	2.825
11-,13-,15-MeC35	0.786	0.702	0.386	0.035	0	0	1.642	0	0	0.734
11,15-diMeC27	0.259	0.764	0.415	0.251	0.714	0.193	1.161	0.557	0.381	0.59
11,15-diMeC29	1.895	3.919	2.24	1.725	3.486	1.839	6.588	2.862	2.713	4.578
12,16-diMeC30	0.34	0	0	0.095	0.332	0.054	0.67	0.267	0.331	0.493
11,15-diMeC31	1.551	2.019	1.259	1.022	1.616	0.795	3.623	1.559	1.6	2.945
11,15- and 9,13-diMeC33	1.578	1.23	0.944	0.693	0.691	0.392	2.83	1.076	1.2	2.733
11,15- and 13,17-diMeC35	0.915	0.461	0.273	0.224	0	0	1.319	0	0	1.299
4,8,12-triMeC28	0.193	0.153	0.091	0.066	0	0	0.307	0.103	0.084	0.286
3,7,11-triMeC31	0.54	0.68	0.417	0.273	0.473	0.251	1.782	0.456	0.641	1.534
9,13,21- and 11,15,23-triMeC33	0	0	0	0	0.09	0	0.356	0.133	0	0.441

**Table A2:** Full dataset of compounds (in µg/500 µL injected sample) obtained by GC-MS analysis for the synthetic alkane mixture (alkane_mix) and the extracts of the powdered zeolite samples loaded with this solution.

Compound	Alkane_Mix	HY80	HY30	HBEA30	NH4ZSM5280	HBEA300	NH4MOR38	NH4ZSM530	NaX2.2	HZSM2245
n-C27	23.543	10.775	10.679	3.185	0.039	7.51	7.856	0.061	8.005	0.192
n-C29	19.683	9.192	8.993	1.877	0.035	6.258	6.692	0.04	6.808	0.077
3-MeC24	0.06	0.022	0.028	0.022	0	0.018	0.006	0	0.008	0
3-MeC25	17.044	7.582	7.542	5.901	2.522	6.024	5.171	2.442	5.607	3.565
3-MeC26	0.16	0.056	0.045	0.074	0.006	0	0.035	0	0.013	0.012
3-MeC27	0.278	0.107	0.089	0.067	0.007	0	0.04	0	0.038	0

**Table A3:** Results of the posthoc tests on the Aitchison transformed data to assess the selectivity of the zeolites between two groups of compounds (Selectivity Group 1 and Selectivity Group 2). The *p*-values are provided from which the significance (*p* < 0.05) can be determined. Column HY80 served as the reference sample, so the p-values are not applicable (NA) for this sample.

Selectivity Group 1	Selectivity Group 2	Ants_ref_4	HY80	HY30	HBEA30	NH_4_ZSM5280	HBEA300	NH_4_MOR38	NH_4_ZSM530	NaX2.2	HZSM2245
n-C_27_ and n-C_29_	**3-MeC_25_ and 3-MeC_27_**	0.14	NA	0.25	0.01	3.63 × 10^−6^	0.31	0.20	2.48 × 10^−6^	0.51	7.71 × 10^−6^
n-alkanes (<n-C_30_)	**monomethylalkanes (<C_30_)**	1.64 × 10^−6^	NA	0.71	9.27 × 10^−4^	1.72 × 10^−4^	0.04	0.99	9.18 × 10^−4^	0.06	1.25 × 10^−5^
n-alkanes (<n-C_30_)	**n-alkanes (≥n-C_30_)**	0.65	NA	0.39	4.96 × 10^−2^	2.70 × 10^−8^	0.88	4.84 × 10^−2^	3.05 × 10^−6^	0.04	1.94 × 10^−7^
dimethylalkanes (<C_30_)	**n-alkanes (<n-C_30_)**	0.02	NA	0.90	0.19	3.34 × 10^−4^	0.78	0.93	4.87 × 10^−2^	0.42	0.003
dimethylalkanes	**monomethylalkanes**	0.52	NA	0.82	0.25	1.08 × 10^−3^	0.73	0.88	0.04	0.85	4.84 × 10^−2^
trimethylalkanes	**n-alkanes**	4.07 × 10^−4^	NA	0.37	0.24	4.55 × 10^−3^	2.41 × 10^−3^	0.30	0.01	0.17	2.49 × 10^−5^
trimethylalkanes	**monomethylalkanes**	0.61	NA	0.40	0.26	7.37 × 10^−8^	6.95 × 10^−4^	0.01	7.16 × 10^−6^	0.56	1.47 × 10^−7^
n-alkanes	**n-alkenes and n-alkadienes**	4.87 × 10^−3^	NA	0.59	0.59	1.58 × 10^−5^	1.6 × 10^−6^	0.79	4.99 × 10^−3^	0.95	4.88 × 10^−5^
